# The pathological features of leukemic cells infiltrating the renal interstitium in chronic lymphocytic leukemia/small lymphocytic lymphoma from a large single Chinese center

**DOI:** 10.1186/s13000-021-01120-4

**Published:** 2021-07-04

**Authors:** Hui Wang, Xiaojuan Yu, Xu Zhang, Suxia Wang, Minghui Zhao

**Affiliations:** 1 Laboratory of Electron Microscopy, Peking University First Hospital, Beijing, 100034 People’s Republic of China; 2 Renal Pathology Center, Institute of Nephrology, Peking University, Beijing, 100034 People’s Republic of China; 3 Department of Nephrology, Peking University First Hospital, Beijing, 100034 People’s Republic of China

**Keywords:** Chronic lymphocytic leukemia/small lymphocytic lymphoma (CLL/SLL), Infiltrating CLL cells, Monoclonal immunoglobulins, Pathological features, Tubulointerstitial injury

## Abstract

**Background:**

Chronic lymphocytic leukemia/small lymphocytic lymphoma (CLL/SLL) is rare in Asians, and patients with CLL/SLL seldomly undergo kidney biopsy. The histopathological features and clinical relevance of tubulointerstitial injury in CLL/SLL have not been extensively characterized. Hence, we attempted to describe the clinical characteristics, renal pathology and clinical outcome of a well-characterized population of CLL/SLL patients with CLL cell infiltration in the renal interstitium from a large single center in China.

**Methods:**

Between January 1st, 2010 and September 31st, 2020, 31946renal biopsies were performed at Peking University First Hospital, and 10 CLL/SLL patients with CLL cell infiltration in the renal interstitium were included. Complete clinical data were collected from these 10 patients, and renal specimens were examined by routine light microscopy, immunofluorescence and electron microscopy.

**Results:**

The extent of the infiltrating CLL cells in patients with CLL/SLL varied among different patients and ranged from 10 to 90% of kidney parenchyma. Six (60%) of 10 patients presented with an extent of infiltrating CLL cells ≥50%. Interestingly, we found that three patients (3/10, 30%) expressed monoclonal immunoglobulins in the infiltrating CLL cells, and special cytoplasmic crystalline structures were found in two of the three patients by electron microscopy for the first time. Severe renal insufficiency (Scr ≥200 μmol/L) was associated with ≥50% interstitial infiltration of CLL cells in the renal interstitium.

**Conclusions:**

The current study confirmed that CLL cells infiltrating the renal interstitium can directly secrete monoclonal immunoglobulins, indicating that the interstitial infiltrating CLL cells possibly cause renal injury directly by secreting monoclonal immunoglobulins in situ. This finding may prove a new clue to elucidate the pathogenetic mechanism of renal injury involved with CLL/SLL.

**Supplementary Information:**

The online version contains supplementary material available at 10.1186/s13000-021-01120-4.

## Background

Chronic lymphocytic leukemia/small lymphocytic lymphoma (CLL/SLL), the most common adult leukemia in the Western countries [1], is infrequent in Asians including China. The age-adjusted incidence rate of CLL/SLL in the United States is 4.4/100,000 per year [2]. In contrast, the rate in Asians is 0.2–0.3/100,000 per year [3, 4]. A population-based study of predominately Han Chinese reported an even lower CLL/SLL incidence of 0.05/100,000 per year [5]. This disease is characterized by the clonal expansion of CD5 + CD23+ B cells in peripheral blood, bone marrow, and the secondary lymphoid tissues [6, 7]. Extramedullary/extranodal manifestations of CLL/SLL are rare. The most commonly involved organs are the skin and central nervous system. Kidneys are rarely involved [7–9]. A study from the Mayo Clinic found a 7.5% incidence of renal insufficiency at the time of CLL/SLL diagnosis in a cohort of over 2000 CLL/SLL patients. Importantly, the presence of kidney disease was independently associated with adverse patient outcomes in CLL/SLL [10], which emphasizes the importance of a detailed exploration of the mechanism of renal insufficiency in CLL/SLL.

In renal complications of CLL/SLL, the mechanism of renal insufficiency in such patients varies. Several studies have described the patterns of glomerular injury, which includes direct glomerular deposition of monoclonal proteins, cryoglobulins, and immune complexes [11, 12]. Previous studies related to CLL cell infiltration of the kidney have been either case reports with a single patient or small case series [13–29], with the largest report including 6 patients [11]. In these studies, the histopathological features of CLL cells infiltrating the renal interstitium were not described in detail, and the mechanism of renal injury with CLL cell infiltration remained unclear.

In this study, we attempted to describe the clinical characteristics, renal pathology and clinical outcome of a well-characterized population of CLL/SLL patients with CLL cell infiltration in the renal interstitium from a large single center at Peking University First Hospital in China. We investigated the pathological features of infiltrating CLL cells in the renal interstitium in detail and tried to explore the possible pathogenesis of renal injury in association with CLL/SLL.

## Methods

### Patients

Between January 1st, 2010 and September 31st, 2020, 31,946 renal biopsies were performed at Peking University First Hospital. Finally, after screening of the renal pathology database, 10 (10/31946, 0.03%) patients with CLL cell infiltration in the renal interstitium were included. CLL/SLL was diagnosed according to the WHO classification [30, 31]. The diagnosis of CLL requires the presence of at least 5 × 10^9^ B lymphocytes/L in the peripheral blood over > 3 months, with evidence of monoclonality plus the expression of CD5, CD19, CD20, and CD23. CLL is distinguishable from SLL by its leukemic appearance. The definition of SLL requires a histological analysis of lymph node biopsy. CLL/SLL of all 10 patients was confirmed before or at the time of the kidney biopsy.

Informed consent was obtained from each patient. The research was in compliance with the Declaration of Helsinki and approved by the ethics committee of Peking University First Hospital.

### Clinical and laboratory assessment during follow-up

Baseline clinical data, including age, sex, medical history, CLL clinical course, CLL-involved organs, proteinuria, hematuria, serum creatinine, complete blood test, serum/urine immunofixation electrophoresis, and treatment, were obtained from the clinical records. Patients were followed up in the outpatient clinic or by telephone.

### Renal histopathology assessment

Renal biopsy was examined by standard direct immunofluorescence, light microscopy and electron microscopy. For light microscopy, biopsy specimens were stained with hematoxylin-eosin, periodic acid Schiff, Masson trichrome, and Jones methenamine silver. For immunofluorescence, 3-μm cryostat sections were stained with polyclonal fluorescein isothiocyanate-conjugated antibodies of the IgG, IgM, IgA, C3, C1q, kappa light chain, lambda light chain and IgG subclasses. Electron microscopy was performed as per routine clinical practice. Immunohistochemical staining for CD3, CD5, CD20, CD23, Cyclin D1, CD43, TdT, CD10, and BCL6 was performed on renal biopsies to assess CLL/SLL involvement. Two pathologists specializing in the evaluation of renal pathology separately evaluated the renal biopsies. All cases were also reviewed by hematopathologists specializing in review of lymphoid malignancy. Differences in diagnosis between the two pathologists were resolved by re**-**reviewing the biopsies to reach a consensus.

### Immunoelectron microscopy

Immunogold labeling was further performed according to the methods described by previous studies [32]. Eighty-nm-thick resin-embedded sections were attached to the surface of a carbon-coated support film grid and blocked with 2% bovine serum albumin in 0.01 M phosphate buffer saline, pH 7.4, saline for 5 min at 37 °C, followed by incubation overnight at 4 °C using an extensive panel of antibodies such as polyclonal rabbit anti-human kappa and lambda light chain (1:1000; Dako, Carpenteria, CA) and monoclonal mouse anti-human IgG1, IgG2, IgG3, and IgG4 (1:100; Southern Biotech, Birmingham, AL). Then, after incubation with a gold-conjugated secondary antibody (colloid gold particles with a diameter of 10 nm) for 1 h, the grid was ready for observation using a transmission electron microscope (JEM-1230, JEOL, Tokyo, Japan). Negative controls were prepared by omitting the primary antibody.

### Statistical analysis

Statistical software SPSS 13.0 (SPSS, Chicago, IL, USA) was used. Descriptive statistics of the baseline characteristics were calculated. Continuous data are expressed as median with the range. Categorical variables are presented as proportions. Correlations between pathological characteristics in the kidney interstitium and serum creatinine at renal biopsy were performed using Fisher’s exact test. Kaplan-Meier curves were used to analyze the patients’renal outcomes.

## Results

### Demographic and baseline clinical data

The clinical features at biopsy are presented in Table 1. Five males (50%) and 5 females (50%) were enrolled. The median age was 66 (range 58–73) years old. CLL/SLL was diagnosed before kidney biopsy in 5/10 cases, with a median interval of 18 months (range 2–60 months). Four patients were biopsied for acute kidney disease, two for acute kidney injury and four for proteinuria. The median serum creatinine at renal biopsy was 197.5 μmol/L (range: 95–616 μmol/L). The median proteinuria was 3.5 g/24 h (range: 0.48–12.8), with a median serum albumin of36.1 g/L (range: 18.1–46.7). Three patients (30%) had nephrotic syndrome or nephrotic range proteinuria. Seven patients (70%) had microscopic hematuria. All patients underwent bone marrow biopsy, and eight patients (80%) showed CLL/SLL cell bone marrow infiltration. Eight patients (80%) had multiple enlarged lymph nodes, and all of them had lymph node biopsy involved by SLL. One patient had splenomegaly. Six patients (60%) had positive serum monoclonal immunoglobulins, including 3 patients with IgMκ, 2 patients with IgGκ and 1 patient with IgMλ, respectively.

**Table 1:** The clinical features of CLL/SLL patients

Case	Age/sex	Time (months)	Scr (μmol/L)	UTP (g/24 h)	MIg	Cryo	C3 (g/L)	C4 (g/L)	ER-IO	Treatment	F. time (months)	Renal outcome	CLL/SLL
1	67/M	2	118	10.35	IgMλ	Trace	0.57↓	0.12	LN	CYC, CsA	56	Not recovery	Stable
2	66/M	0	616	0.80	Negative	Trace	0.63	0.26	LN, BM	Supportive	52	ESRD (never recovered)	Stable
3	58/F	0	95	1.15	IgGκ	Negative	0.92	0.21	BM	COP, Rituximab Ibrutinib	32	Completely recovery	Improved
4	58/F	0	174	0.48	IgMκ	Negative	0.48↓	0.05↓	LN,BM	RFC	31	Completely recovery	Stable
5	66/F	60	570	0.87	Negative	NA	0.72	0.34	LN, Spleen BM	CHOP, Ibrutinib	15	Partial recovery	Stable
6	62/M	36	118	4.27	IgGκ	IgGκ	0.57↓	0.09↓	LN,BM	COP, Ibrutinib	8	Completely recovery	Stable
7	68/F	18	456	2.17	Negative	NA	0.81	0.16	LN,	Prednisone	6	Partial recovery	Improved
8	59/F	0	377	0.59	IgMκ	Type II	0.46↓	0.02↓	LN,BM	PE for TMA	33	Partial recovery	Progressed
9	73/M	3	161	1.44	Negative	Negative	0.76	0.20	BM	Supportive	2	Not recovery	Stable
10	67/M	0	221	12.8	IgMκ	IgMκ	0.244↓	0.06↓	LN,BM	Ibrutinib	1	Partial recovery	Stable

Notes: Time: time from diagnosis of CLL/SLL to renal biopsy; Scr: serum creatinine at renal biopsy

Abbreviations: *UTP* urine total protein, *MIg* monoclonal immunoglobulin determined by serum/urine immunofixation electrophoresis; *Cryo* cryoglobulinemia, *ER-IO* extra-renal involved organs, *F.time* follow up time, *LN* lymph node, *BM* bone marrow, *CYC* cyclophosphamide, *CsA* cyclosporine, *ESRD* end stage renal disease, *COP* cyclophosphamide, vincristine, and prednisone, *RFC* rituximab, fludarabine and cyclophosphamide, *CHOP* cyclophosphamide, doxorubicin, vincristine and prednisone, *PE* plasma exchange, *TMA* thrombotic microangiopathy

### Renal biopsy findings and characteristics of CLL cell infiltration in the renal interstitium

**Fig. 1:**
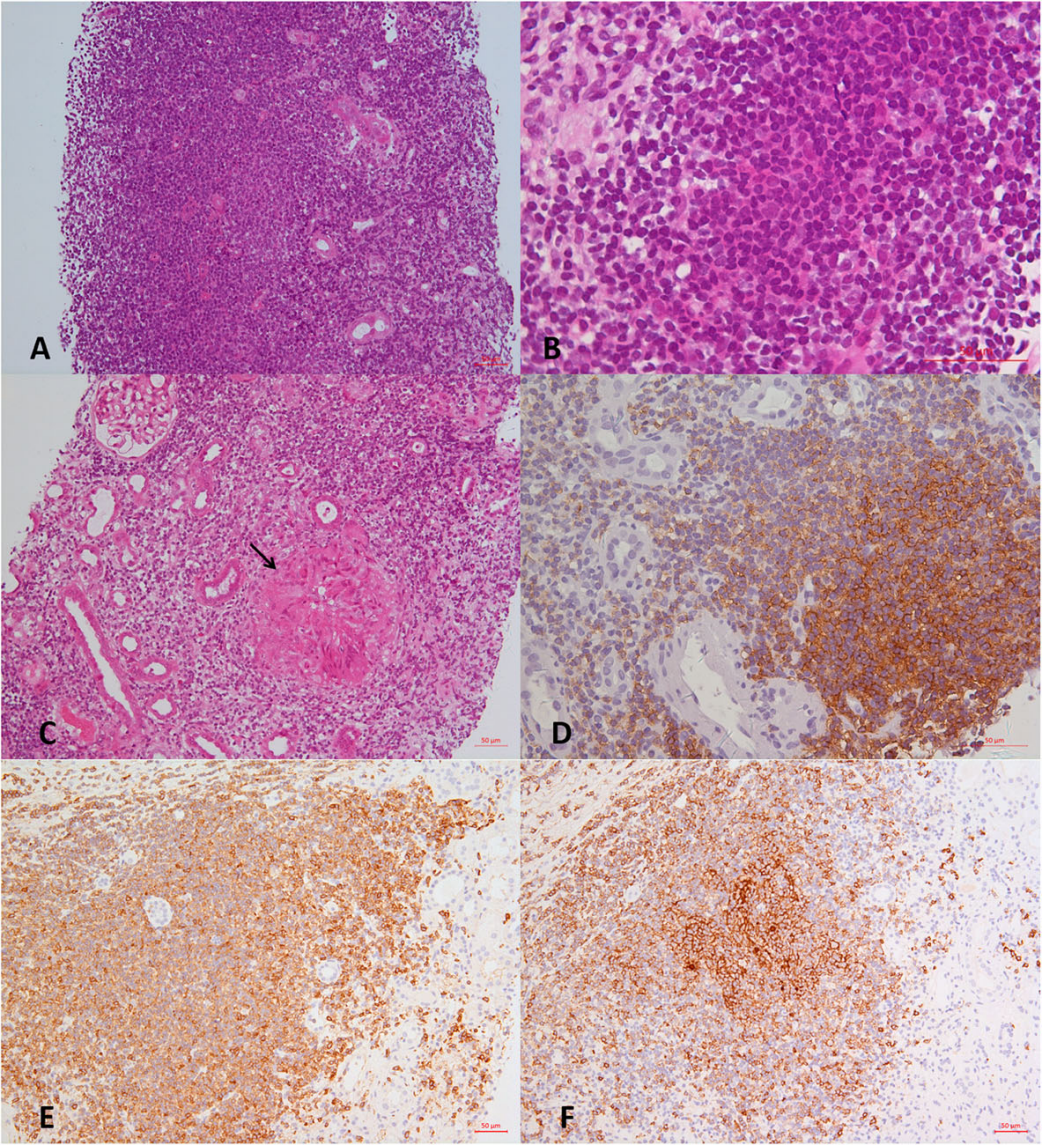
Representative light microscopic findings of renal biopsy. **A**. CLL cells infiltration was seen in the renal interstitium (HE, ×200). **B**. Vaguely nodular pattern was seen in the renal interstitium(HE, × 630). **C**. Interstitial epithelioid granulomas were formed in the renal interstitium as indicated by the arrow (HE, × 200). **D**. Infiltrating CLL cells stain positively for CD20 (Immunohistochemistry, × 400). **E**. Infiltrating CLL cells stain positively for CD5 (Immunohistochemistry, × 200). **F**. Infiltrating CLL cells stain positively for CD23 (Immunohistochemistry, × 200)

Table 2 summarizes the kidney biopsy pathology and characteristics of the interstitial infiltrating CLL cells. All of these patients presented varying degrees of monotypic small lymphocyte infiltration in the renal interstitium. These changes expanded the interstitium at the expense of the tubular structures, the peritubular capillaries, and less frequently the glomeruli (Fig. 1A). Immunohistochemical staining showed the monotypic lymphocyte cells stained positive for CD20 (Fig. 1D), CD5 (Fig. 1E) and CD23 (Fig. 1F) and negative for Cyclin D1, CD10, CD138 and CD68 .

**Table 2:** The kidney biopsy pathology and characteristics of the interstitial infiltrating CLL cells

Case	Glomerular			Interstitium				
	Light microscopy	Immunofluorescence	Electron microscopy	Light microscopy			Immunofluo-rescence	Electron microscopy
	Injury pattern		Electron dense deposits	The extent of infiltrate CLL cells	Nodular pattern formation	Granulomatous		
1	Membranous nephritis	IgG++,C3+, C1q+	Subepithelial deposits	10%	No	No	Negative	No remarkable change
2	Minor change	Negative	No deposits	90%	Yes	No	Negative	Infiltrating neoplastic cells with crystal formation
3	Focal mesangial and endocapillary proliferative glomerulonephritis	C3++	Mesangial and hump-like subepithelial deposits	10%	No	No	Negative	No remarkable change
4	Membranoproliferative glomerulonephritis	IgG++,IgM+++,C3+,C1q+,κ++,IgG1++,IgG2++	Subendothelial deposits with microtubule formation	10%	No	No	Negative	No remarkable change
5	Minor change	Negative	No deposits	90%	Yes	Yes	Negative	No remarkable change
6	Membranoproliferative glomerulonephritis	IgG++,C3++,C1q+,κ++IgG1++	Subendothelial and subepithelial deposits with microtubule formation	70%	Yes	No	κ++IgG1++	Infiltrating neoplastic cells with microtubule formation
7	Focal mesangial and endocapillary proliferative glomerulonephritis	C3++	Mesangial and hump-like subepithelial deposits	50%	Yes	No	Negative	No remarkable change
8	Mesangial proliferative glomerulonephritis and thrombotic microangiopathy	C3++	Mesangial and Subendothelial deposits	50%	Yes	No	Negative	No remarkable change
9	Glomerular hypertrophy	Negative	No deposits	20%	No	No	Negative	No remarkable change
10	Membranoproliferative glomerulonephritis	IgM++,κ++	Subendothelial and mesangial deposits with microtubule formation	50%	Yes	No	IgM++,κ++	No remarkable change

**Fig. 2:**
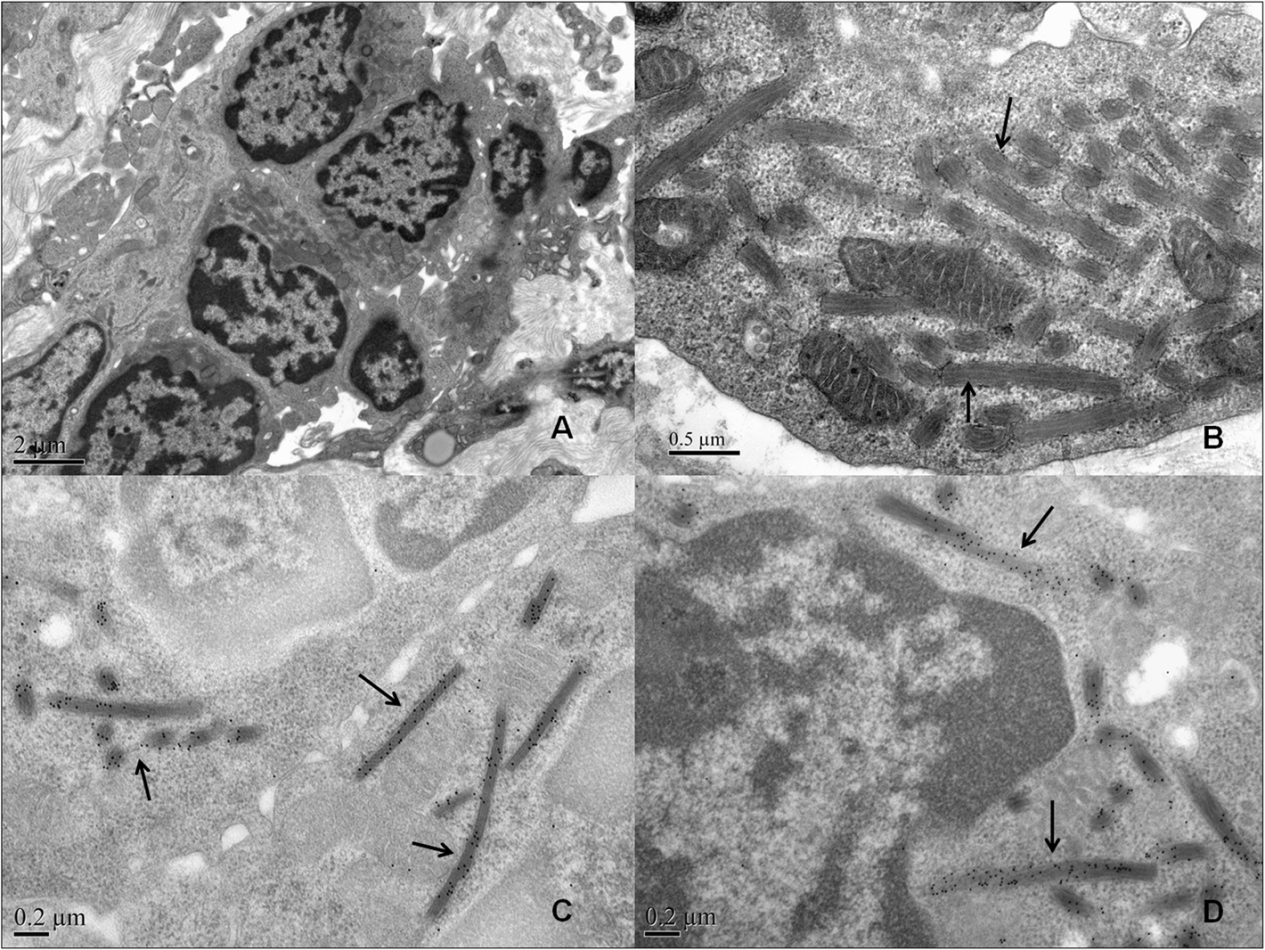
Representative electron microscopy findings of renal biopsy in case 6. **A**. Neoplastic cells infiltration was seen in the renal interstitium (×10,000). **B**. Special cytoplasmic crystals characterized by fuzzy filament structure were found in infiltrating neoplastic cells as indicated by arrows (×40,000). **C**. Intense IgG1 was detected in the crystalline structures of neoplastic cells as indicated by the arrow (Immunoelectron microscopy, ×50,000). **D**. Intense kappa was detected in the crystalline structures of neoplastic cells as indicated by the arrow (Immunoelectron microscopy, × 50,000)

**Fig. 3:**
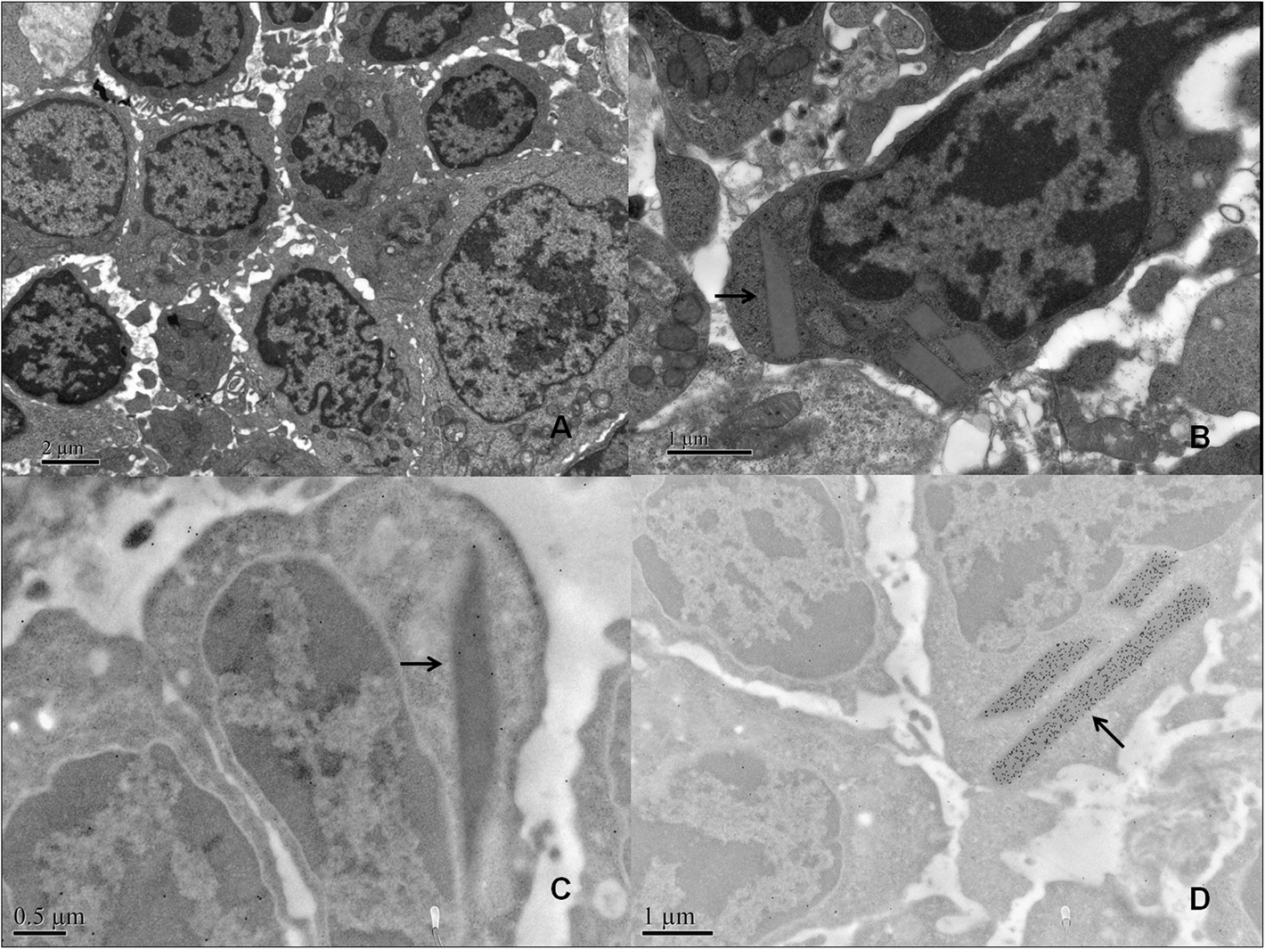
Representative electron microscopy findings of renal biopsy in case 2. **A**. Neoplastic cells infiltration was seen in the renal interstitium (× 8000). **B**. Rhombic crystals were formed in infiltrating neoplastic cells as indicated the arrow (×25,000). **C**. Light kappa was not detected in neoplastic cells (Immunoelectron microscopy labelling, ×30,000). **D**. Intense light lamda was detected in the crystalline structures of neoplastic cells as indicated by the arrow (Immunoelectron microscopy labelling, × 20,000)

The extent of infiltrating CLL cells was diverse between different patients, from 10 to 90%. Six patients (60%) presented interstitial CLL cell infiltration ≥50%. Vaguely nodular pattern can be seen among infiltrating CLL cells of these 6 patients (Fig. 1B), in which prolymphocytes and paraimmunoblasts were detected. Notably, of the 6 patients, two patients (Case 6 and Case 10) showed monoclonal immunoglobulin expression in the renal interstitium by immunofluorescence (IgG1κ for Case 6 and IgMκ for Case 10), which was identical to that of the serum immunoglobulin paraprotein (Supplementary Fig. 1). By electron microscopy observation, special crystalline structures were found in the cytoplasm of infiltrating CLL cells in two patients (Case 2 and Case 6). In Case 6, a fuzzy filament structure was observed in these crystalline structures (Fig. 2), and IgG1κ expression was detected in these crystals by immunoelectron microscopy (Fig. 2). In another patient (Case 2), although immunofluorescence showed no specific interstitial deposits, rhombic crystals were also found in the cytoplasm of infiltrating CLL cells, and monoclonal λ was expressed in these crystals by immunoelectron microscopy (Fig. 3). In brief, three patients (30%) expressed monoclonal immunoglobulins in the infiltrating CLL cells and the cytoplasmic crystals were identified in two patients (20%). In addition, non-necrotic epithelioid granulomas were found in one patient (Case 5, Fig. 1). Periodic acid Schiff and Grocott staining revealed no microorganisms. Immunofluorescence showed no specific interstitial deposits.

Eight patients (80%) also had concurrent glomerular diseases. Membranoproliferative glomerulonephritis (MPGN) was present in 3 patients (Cases 4, 6 and 10). All of them showed glomerular deposits composed of monoclonal immunoglobulins (IgMκ in 2 cases, IgGκ in one case) by immunofluorescence. Two patients exhibited patterns of endocapillary proliferative glomerulonephritis (Cases 3 and 7). The glomeruli exhibited endocapillary hypercellularity with infiltration of neutrophils. Immunofluorescent microscopy showed coarse granular staining of C3 along the glomerular capillary loops without “masked” immune deposits identified by immunohistochemical detection on paraffin sections with protease digestion. Massive hump-like subepithelial deposits were also found under electron microscopy. These findings indicated probable bacterial infection-associated glomerulonephritis in these two patients. The other glomerular diseases included one patient with phospholipase A2 receptor-negative membranous nephropathy (Case 1), one thrombotic microangiopathy concomitant with mesangial proliferative C3 glomerulonephritis (Case 8), and one obesity-associated glomerular hypertrophy (Case 9) respectively.

### Correlation between pathological characteristics and serum creatinine at renal biopsy

Next, we assessed whether pathological characteristics affected renal function at renal biopsy. There were 5 patients whose Scr was ≥200 μmol/L at renal biopsy and 5 patients with < 200 μmol/L. The Scr (≥200 μmol/L) was associated with interstitial fibrosis and tubular atrophy (IFTA) (≥50%) (*P* = 0.048), CLL cell infiltration (≥50%) (*P* = 0.048), and nodular pattern formation (*P* = 0.048) (Supplementary Table 1). Pathology characteristics of glomeruli including global glomerulosclerosis, mesangial proliferation, endothelial proliferation and crescents were not associated with severe renal failure (Scr ≥200 μmol/L).

Then, we assessed the correlations between pathological characteristics. CLL cell infiltration (≥50%) was significantly correlated with nodular pattern formation (*P* = 0.005). A borderline significant association was also found between CLL cell infiltration (≥50%) and IFTA (*P* = 0.076). In addition, IFTA was significantly correlated with global glomerulosclerosis (*P* = 0.033). No other associations between pathological characteristics was found.

### Treatment and renal outcome

The mean follow-up time was 23.6 months (range 1–56). Five patients (Case 3, 4, 5, 6 and 10) received chemotherapy (COP, CHOP, RFC) and/or molecular targeted therapy (Rituximab, Ibutinib) (Table 1). Two patients received prednisone (Cases 7) or immunosuppressant therapy (Cases 1). The remaining three patients only received supportive treatment. All patients who received chemotherapautic drugs or molecular targeted therapy achieved completely remission (Cases 3, 4 and 6) or partial remission (Cases 5 and 10). In two patients without concomitant glomerular disease, only one patient (Cases 5) received chemotherapautic and molecular targeted therapeutic drugs therapy and the renal outcome achieved partial recovery. However, the other one (Cases 2) who only received supportive treatment developed end stage renal disease.

### Correlation between clinicopathological characteristics and renal outcome

For the univariate survival analysis of the renal outcome, no significant differences were found between renal outcome and clinicopathological characteristics, including serum creatinine, global glomerulosclerosis, mesangial proliferation, endothelial proliferation, crescents, IFTA, CLL cell infiltration and treatment (data not shown).

## Discussion

CLL/SLL is extremely rare in Asians compared with persons of predominately European descent [1–5]. Unlike plasma cell dyscrasias, in which kidney biopsy is routinely performed to evaluate unexplained renal insufficiency [33], patients with CLL/SLL rarely undergo kidney biopsy because CLL/SLL usually follows an indolent course. In the series from the Mayo Clinic [11], of all the CLL/SLL patients studied, only 1.2% underwent kidney biopsy. The low rate of kidney biopsy is a limiting factor in our understanding of CLL/SLL-associated kidney disease. Here, we attempted to better describe the pathological features of CLL cells infiltrating in the renal interstitium of CLL/SLL patients in a Chinese cohort. In particular, we found that infiltrating CLL cells expressed monoclonal immunoglobulins with crystal formation for the first time. These pathological features suggested that in situ secretion of monoclonal immunoglobulins by renal infiltrating CLL cells may contribute to the pathogenetic mechanism of renal injury in patients with CLL/SLL.

Renal interstitial infiltration is a frequent finding in autopsy series of CLL/SLL patients, and the extent of infiltrating CLL cells was estimated to be between 10 and 90% in previous reports [13–29]. However, the specific role of these infiltrating CLL cells in the development and progression of renal disease is equivocal. To date, 37 cases with renal failure due to CLL cells’ infiltration have been described in the literature, including 2 cases in our study. In our study, severe renal failure (Scr ≥ 200 μmol/L) at renal biopsy was associated with interstitial fibrosis and tubular atrophy (IFTA) (≥50%), diffuse CLL cell infiltration (≥50%) and nodular pattern formation. Although the sample size was not sufficiently large in our study, the significant association indicated that diffuse infiltrating CLL cells (≥50%) might affect the severity of kidney failure at presentation. Although 8/10 patients had concomitant glomerular disease which may be the reason for biopsy or major contributor to the kidney disease, the associations between pathology characteristics of glomerular and severe renal failure (Scr ≥ 200 μmol/L) showed no statistically significant in our study, which may be due to the heterogeneity of the glomerular diseases. Whether diffuse CLL cells’ infiltration by itself carries a worse prognosis cannot be answered in our analysis given the limited sample size. Nonetheless, the borderline significant association between diffuse CLL cells’ infiltration and IFTA suggested diffuse CLL cells’ infiltration may influence renal prognosis, for IFTA has been demonstrated as an independent risk factor for renal outcome in many kidney diseases, such as lupus nephritis and IgA nephropathy [34, 35]. In addition, in two patients without concomitant glomerular disease, the one who received chemotherapautic and molecular targeted therapeutic drugs therapy achieved partial recovery of renal outcome. However, the other one who only received supportive treatment developed end stage renal disease. This also suggested CLL infiltration in the kidney possibly caused renal injury, which should be paid more attention to.

To date, the mechanism of renal injury with CLL cell infiltration has not been clearly established but has been hypothesized to involve tubular/microvascular compression causing intrarenal obstruction in addition to an infiltration-associated inflammatory/cytokine response [11, 12]. For example, diffuse infiltration likely compresses the renal tubules and microvasculature, resulting in intrarenal obstruction and ischemia. Alternatively, cytokines such as IL-1, IL-6, TNF-α, and TGF-β, secreted directly by lymphoma cells, may lead to tubular injury and interstitial fibrosis [36]. Interestingly, we found that infiltrating CLL cells expressed monoclonal immunoglobulins with crystal formation for the first time, which suggested that CLL cells are possibly directly involved in local injuries by secreting monoclonal immunoglobulins in situ. The presence of serum monoclonal immunoglobulin has been shown to worsen the prognosis of CLL/SLL patients [37, 38]. Although it was not clear whether monoclonal immunoglobulins secreted by local infiltrating CLL cells can enter the blood circulation, the presence of cytoplasmic crystal formation was sufficient to cause cell injury. For example, when crystals of monoclonal light chains deposit in the cytoplasms of proximal tubules, patients exhibit features of proximal tubular dysfunction called light chain proximal tubulopathy (LCPT) [39]. Previous studies showed that some myeloma light chains were toxic to cultured human proximal tubule cells and induced cytoskeletal injury and DNA damage followed by secondary cell necrosis [40]. Myeloma light chains also induced epithelial-mesenchymal transition through p38 MAPK in human renal proximal tubule epithelial cells, which can contribute to IFAT [41]. Therefore, it is possible that monoclonal immunoglobulins secreted by CLL cells in the renal interstitium may contribute to the injury in situ directly.

Moreover, nodular pattern was seen among infiltrating CLL cells in the renal interstitium and prolymphocytes and paraimmunoblasts were detected. A histologic hallmark of CLL/SLL, when it involves lymph nodes, is the formation of proliferation centers characterized by nodular expansions of prolymphocytes and paraimmunoblasts admixed with small lymphocytes. Our study indicated that this organizational structure also formed in the renal interstitium. Recent studies suggest a correlation between a more aggressive disease and proliferation centers in both lymph nodes and bone marrow and suggest that extended proliferation centers may represent a new prognostic marker of CLL/SLL [42–44]. These findings were consistent with our study showing that nodular pattern was associated with severe renal failure (Scr ≥ 200 μmol/L) at renal biopsy. An increasing body of data suggests that proliferation centers are important sites of cell proliferation and the accumulation of genomic aberrations [45]. Proliferation center cells express proteins associated with increased levels of cell proliferation-related markers, including Ki67, CD23, CD71, MUM1/IRF-4 and cyclin D1 [46, 47]. Interactions between CLL and accessory cells within proliferation centers are critical for providing growth and survival signals to CLL B cells, inducing their proliferation, promoting differentiation into either an antibody-secreting cell or a memory cell, or maintaining a nonsecreting blast [48]. Our study indicated that the kidney microenvironment may play a central role in the pathogenesis of CLL. More therapeutic efforts need to be made to disrupt the crosstalk between CLL cells and signals of the microenvironment in the kidney interstitium in the future.

## Conclusions

In summary, we attempted to better describe the pathological features of CLL/SLL patients with CLL cell infiltration in the renal interstitium in a Chinese cohort. Notably, monoclonal immunoglobulins were secreted by CLL cells infiltrating the renal interstitium with crystal formation, indicating that these cells are possibly directly involved in local injuries by secreting monoclonal immunoglobulins in situ. This finding may prove a new clue to elucidate the pathogenetic mechanism of renal injury involved with CLL/SLL, thereby improving our current therapeutic efforts.

## Supplementary Information

**Additional file 1: Supplementary Table 1.** Association between serum creatinine and pathological characteristics at the time of renal biopsy. **Supplementary Figure 1.** A. Scattered granular staining of IgG1 was shown in the interstitium of Case 6 as indicated by the arrow with crumby staining in glomeruli indicated by the arrowhead (Immunofluorescence staining,× 200). B. Scattered granular staining of κ of Case 6 was shown in the interstitium as indicated by the arrow with crumby staining in glomeruli indicated by the arrowhead (Immunofluorescence staining,× 200). C. Scattered granular staining of IgM was shown in the interstitium of Case 10 as indicated by the arrow (Immunofluorescence staining,× 200). D. Scattered granular staining of κ was shown in the interstitium of Case 10 as indicated by the arrow (Immunofluorescence staining,× 200).

## Data Availability

All data generated or analysed during this study are included in this published article.

## Abbreviations

CLL: Chronic lymphocytic leukemia
SLL: Small lymphocytic lymphoma
MPGN: Membranoproliferative glomerulonephritis
IFTA: Interstitial fibrosis and tubular atrophy
LCPT: Light chain proximal tubulopathy
