# Supplementary material for: The pathological features of leukemic cells infiltrating the renal interstitium in chronic lymphocytic leukemia/small lymphocytic lymphoma from a large single Chinese center
Source: Diagn Pathol. 2021 Jul 4;16:59. doi: 10.1186/s13000-021-01120-4 (PMC8254985; doi:10.1186/s13000-021-01120-4)
Supplement: Supplementary file 1 — Additional file 1: Supplementary Table 1. Association between serum creatinine and pathological characteristics at the time of renal biopsy. Supplementary Figure 1. A. Scattered granular staining of IgG1 was shown in the interstitium of Case 6 as indicated by the arrow with crumby staining in glomeruli indicated by the arrowhead (Immunofluorescence staining,× 200). B. Scattered granular staining of κ of Case 6 was shown in the interstitium as indicated by the arrow with crumby staining in glomeruli indicated by the arrowhead (Immunofluorescence staining,× 200). C. Scattered granular staining of IgM was shown in the interstitium of Case 10 as indicated by the arrow (Immunofluorescence staining,× 200). D. Scattered granular staining of κ was shown in the interstitium of Case 10 as indicated by the arrow (Immunofluorescence staining,× 200). [file 13000_2021_1120_MOESM1_ESM.doc]

**Supplementary table 1 Association between serum creatinine and pathological characteristics at the time of renal biopsy**

|  |  | Scr<200 μmol/L | Scr≥200 μmol/L | *P^a^* |
| --- | --- | --- | --- | --- |
| Concomitant glomerular diseases | No | 0 (0) | 2 (40) | 0.444 |
|  | Yes | 5 (100) | 3 (60) |  |
| Global glomerulosclerosis | <20% | 5 (100) | 2 (40) | 0.167 |
|  | ≥20% | 0 (0) | 3 (60) |  |
| Crescents | No | 3 (60) | 2 (40) | 1.000 |
|  | Yes | 2 (40) | 3 (60) |  |
| Mesangial proliferation | No | 2 (40) | 2 (40) | 1.000 |
|  | Yes | 3 (60) | 3 (60) |  |
| Endothelial proliferation | No | 1 (20) | 3 (60) | 0.524 |
|  | Yes | 4 (80) | 2 (40) |  |
| Interstitial fibrosis and tubular atrophy (IFTA) | <50% | 5 (100) | 1 (20) | **0.048** |
|  | ≥50% | 0 (0) | 4 (80) |  |
| CLL cell infiltration | <50% | 4 (80) | 0 (0) | **0.048** |
|  | ≥50% | 1 (20) | 5 (100) |  |
| CLL cells with nodular pattern | No | 4 (80) | 0 (0) | **0.048** |
|  | Yes | 1 (20) | 5 (100) |  |
| Monoclonal immunoglobulins in CLL cells | No | 4 (80) | 3 (60) | 1.000 |
|  | Yes | 1 (20) | 2 (40) |  |
| Granulomatous | No | 5 (100) | 4 (80) | 1.000 |
|  | Yes | 0 (0) | 1 (20) |  |

^a^*P* value estimated in Fisher's exact test.


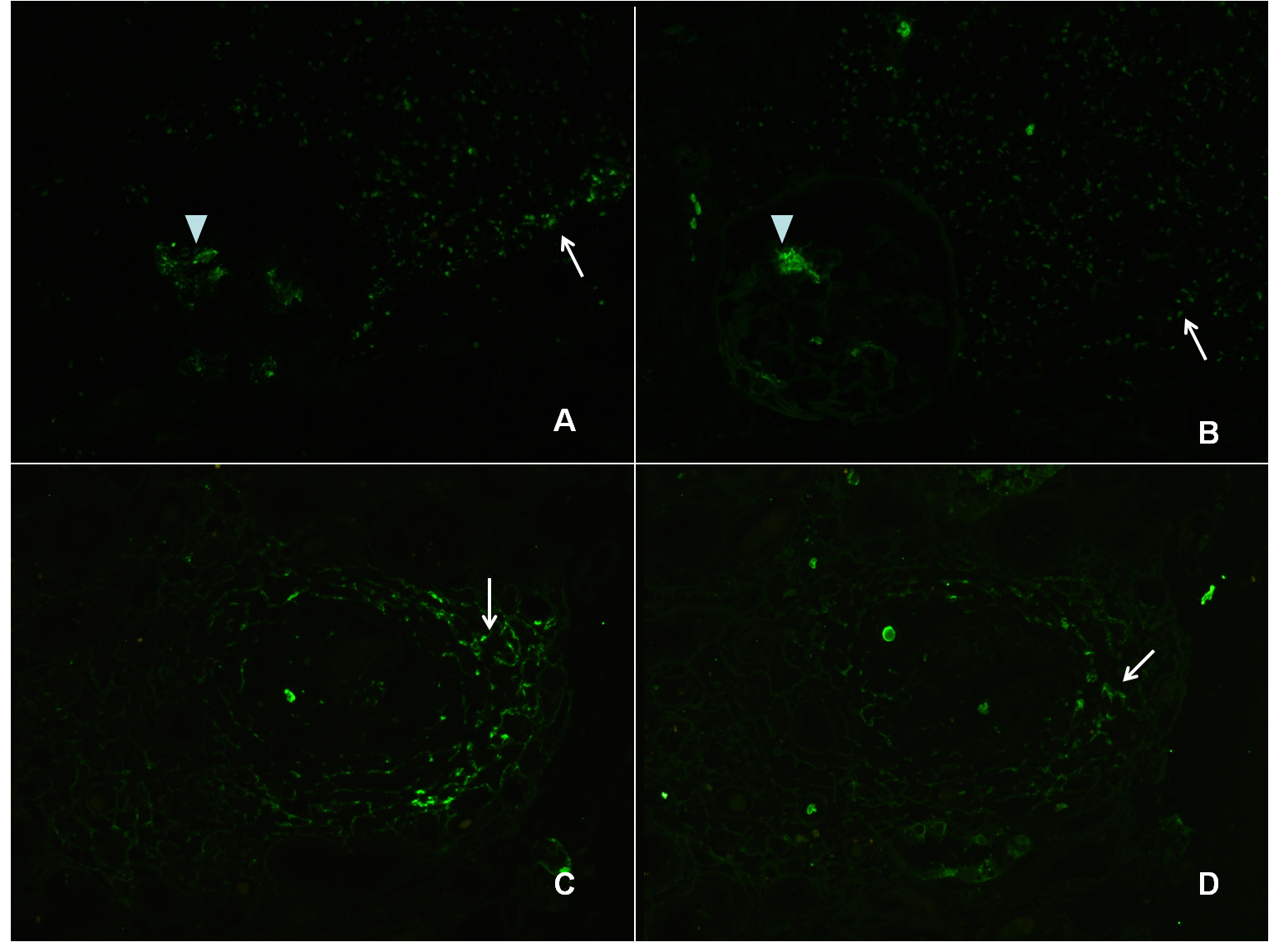


**Supplementary Figure 1.** A. Scattered granular staining of IgG1 was shown in the interstitium of Case 6 as indicated by the arrow with crumby staining in glomeruli indicated by the arrowhead (Immunofluorescence staining,×200). B. Scattered granular staining of κ of Case 6 was shown in the interstitium as indicated by the arrow with crumby staining in glomeruli indicated by the arrowhead (Immunofluorescence staining,×200). C. Scattered granular staining of IgM was shown in the interstitium of Case 10 as indicated by the arrow (Immunofluorescence staining,×200). D. Scattered granular staining of κ was shown in the interstitium of Case 10 as indicated by the arrow (Immunofluorescence staining,×200).
